# EF-P inhibits ribosomal α-hydroxy acid incorporation: strategic tRNA body selection for co-incorporating α-hydroxy acids and nonproteinogenic amino acids into depsipeptides

**DOI:** 10.1261/rna.081083.126

**Published:** 2026-09

**Authors:** Takayuki Katoh

**Affiliations:** Department of Chemistry, Graduate School of Science, The University of Tokyo, Tokyo 113-0033, Japan

**Keywords:** EF-P, α-hydroxy acids, ribosomal ester bond formation, depsipeptides, tRNA body selection, D-arm

## Abstract

Genetic code reprogramming has enabled the ribosomal incorporation of diverse nonproteinogenic amino acids (npAAs) into nascent peptide chains. While backbone-altering npAAs—such as β-amino, γ-amino, d-amino, and *N*-methylamino acids—are typically poor substrates, the development of engineered tRNAs, notably tRNA^Pro1E2^, has facilitated their efficient incorporation. tRNA^Pro1E2^ possesses a distinct D-arm motif recognized by the translation factor EF-P, which enhances peptide bond formation with these npAAs. α-Hydroxy acids can also be ribosomally incorporated to form ester bonds, serving as valuable building blocks for novel bioactive depsipeptides. In this study, α-hydroxy acid incorporation was unexpectedly found to be significantly inhibited by EF-P when charged onto EF-P-responsive tRNAs, such as tRNA^Pro1^ and tRNA^Pro1E2^. This inhibition was effectively disrupted by a single mutation in the D-arm motif. To enable the efficient co-incorporation of α-hydroxy acids and other backbone-altering npAAs (e.g., β- and d-amino acids) in a single translation, a dual-tRNA strategy was employed: α-hydroxy acids were charged onto an EF-P-insensitive tRNA (tRNA^AsnE2^), while β-/d-amino acids were charged onto tRNA^Pro1E2^. Using this approach, a model depsipeptide containing two α-hydroxy acids, one β-amino acid, and one d-amino acid was successfully synthesized in an EF-P-dependent manner.

## INTRODUCTION

In nature, the ribosomal translation system exclusively employs the 20 proteinogenic amino acids (pAAs). Advances in genetic code reprogramming, however, have enabled the ribosomal incorporation of a wide range of nonproteinogenic amino acids (npAAs) into peptides and proteins (Noren et al. 1989; Forster et al. 2003). Among these, l-α-amino acids bearing side chain modifications are relatively efficient substrates. In contrast, npAAs with backbone modifications—such as β-amino, γ-amino, d-amino, and *N*-methylamino acids—are generally far less efficient for ribosomal incorporation (Kawakami et al. 2008; Fujino et al. 2013, 2016; Katoh and Suga 2020b). Given that these backbone-altering npAAs represent valuable building blocks for developing novel bioactive artificial peptides and proteins, strategies to enhance their incorporation efficiency have been pursued for over two decades (Kofman et al. 2021; Sigal et al. 2024).

One primary cause of their low incorporation efficiency is the slow peptidyl transfer reaction between npAAs. In nature, the translation factor EF-P accelerates peptidyl transfer between consecutive proline (Pro) residues (Doerfel et al. 2013; Ude et al. 2013). Because Pro-Pro peptide bond formation occurs much more slowly than that of other pAAs, EF-P is essential to prevent ribosomal stalling. I previously demonstrated that EF-P recognizes the specific D-arm structure of P-site peptidyl-prolyl-tRNA^Pro^, characterized by a 9 nt D-loop and a stable 4 bp D-stem (Katoh et al. 2016). It has also been shown that EF-P promotes the ribosomal incorporation of not only Pro but also various backbone-altering npAAs—such as β-amino, γ-amino, and d-amino acids—when they are precharged onto specific tRNAs containing this D-arm motif, such as tRNA^Pro1^, tRNA^Pro1E2^, and tRNA^iniP^ (Katoh et al. 2017, 2020; Katoh and Suga 2018, 2020a,b, 2021, 2023). tRNA^Pro1^ is one of the three *Escherichia coli* isoacceptor tRNAs for Pro, while tRNA^Pro1E2^ is an engineered variant of tRNA^Pro1^ with a substituted T-stem (from tRNA^Glu^) to enhance EF-Tu binding and accelerate A-site accommodation of aminoacyl-tRNA (Katoh et al. 2017). The use of these tRNAs alongside EF-P has facilitated the ribosomal synthesis of diverse model peptides containing multiple or consecutive backbone-altering npAAs, highlighting the utility of EF-P-assisted translation.

The ribosome can also catalyze ester bond formation when a hydroxy acid is precharged onto a tRNA in place of an amino acid (Fahnestock and Rich 1971; Ohta et al. 2007). α-Hydroxy acids are analogs of α-amino acids where the α-amino group is replaced by an α-hydroxy group, which serves as a nucleophile for ester bond formation. Consequently, these substrates enable the ribosomal synthesis of polyesters or polyester/polypeptide hybrids. Exploring whether EF-P could influence the efficiency of hydroxy acid incorporation would provide valuable insights into developing methodologies for the synthesis of these molecules. However, to the best of our knowledge, the effect of EF-P on ester synthesis using hydroxy acids charged onto tRNAs bearing the EF-P-responsive D-arm motif has not yet been evaluated.

## RESULTS

### Inhibition of α-hydroxy acid incorporation by EF-P

**FIGURE 1.**
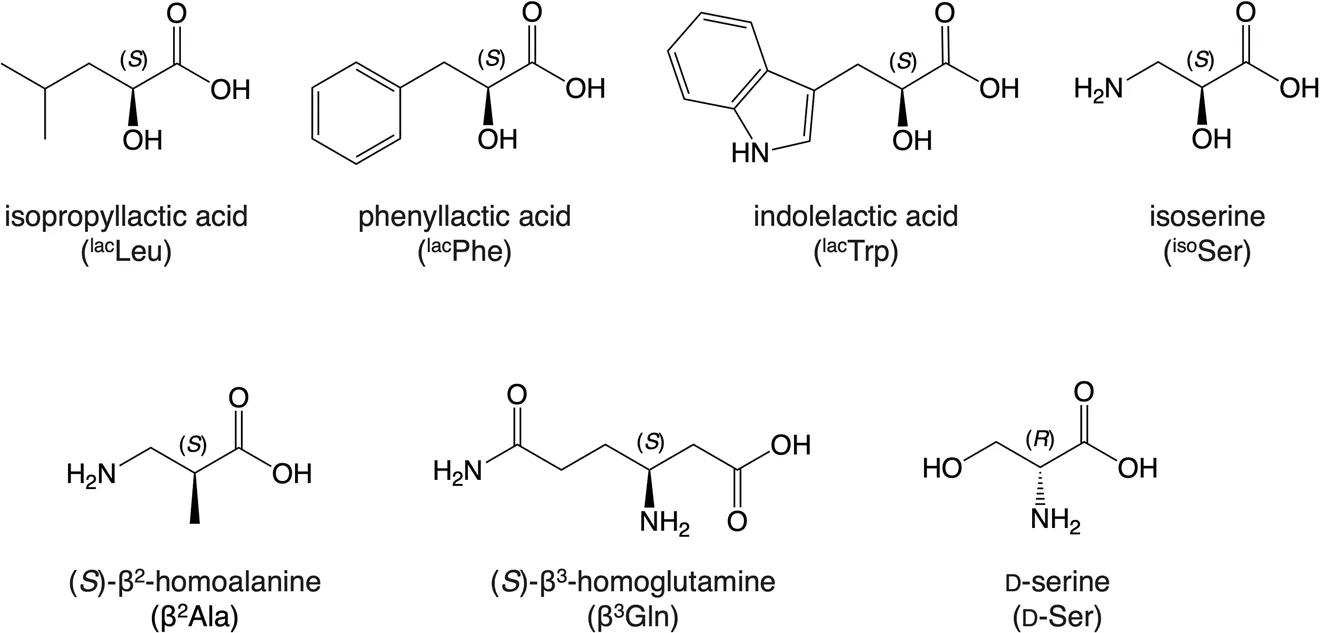
Chemical structures of α-hydroxy acids, β-amino acids, and d-amino acids used in this study.

**FIGURE 2.**
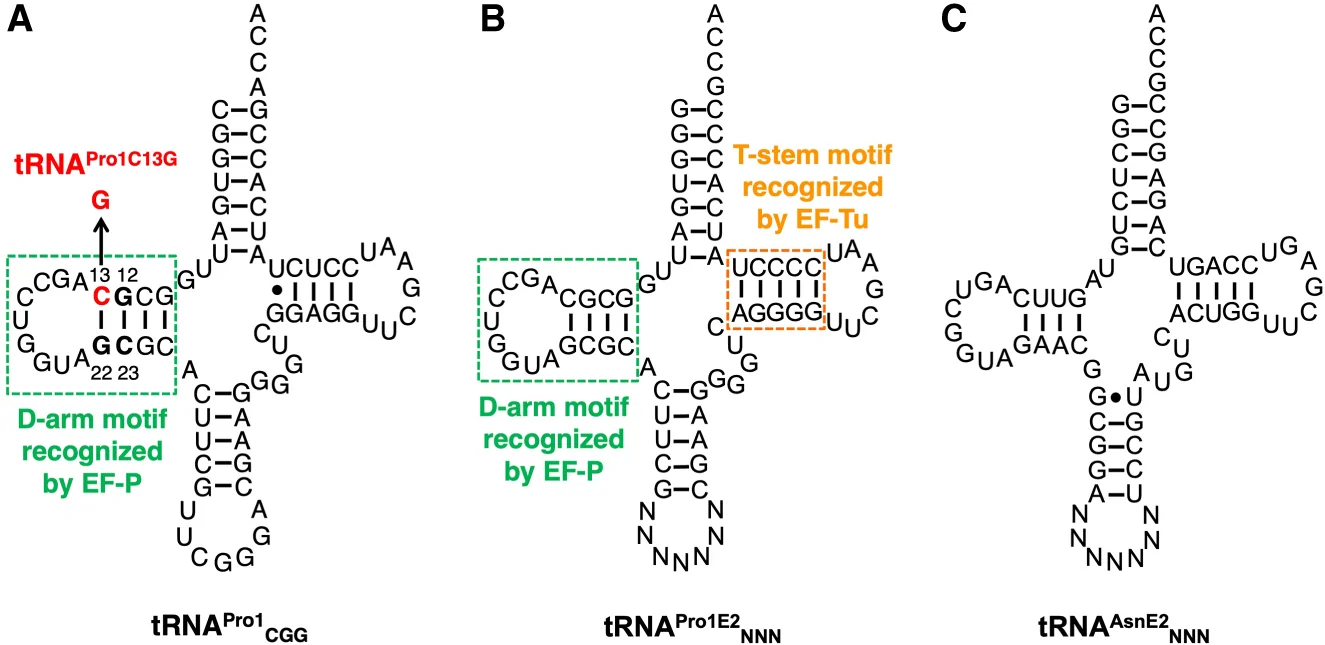
Secondary structures of tRNAs used in this study. (*A*) tRNA^Pro1^_CGG_, (*B*) tRNA^Pro1E2^_NNN_, (*C*) tRNA^AsnE2^_NNN_. The anticodon loop sequences vary depending on the corresponding codon sequences; thus, those of tRNA^Pro1E2^ and tRNA^AsnE2^ are not specified here and are indicated by “NNN.” See Supplemental Table S1 for the specific anticodon loop sequences for tRNA^Pro1E2^ and tRNA^AsnE2^. The D-arm and T-stem motifs recognized by EF-P and EF-Tu are indicated by green and orange boxes, respectively. Substitution of C13 in tRNA^Pro1^ with G (red) yields a mutant tRNA, tRNA^Pro1C13G^. Because G–C base-pairing at positions 13–22 is critical for EF-P binding to the D-arm, tRNA^Pro1C13G^ is significantly less responsive to EF-P.

**FIGURE 3.**
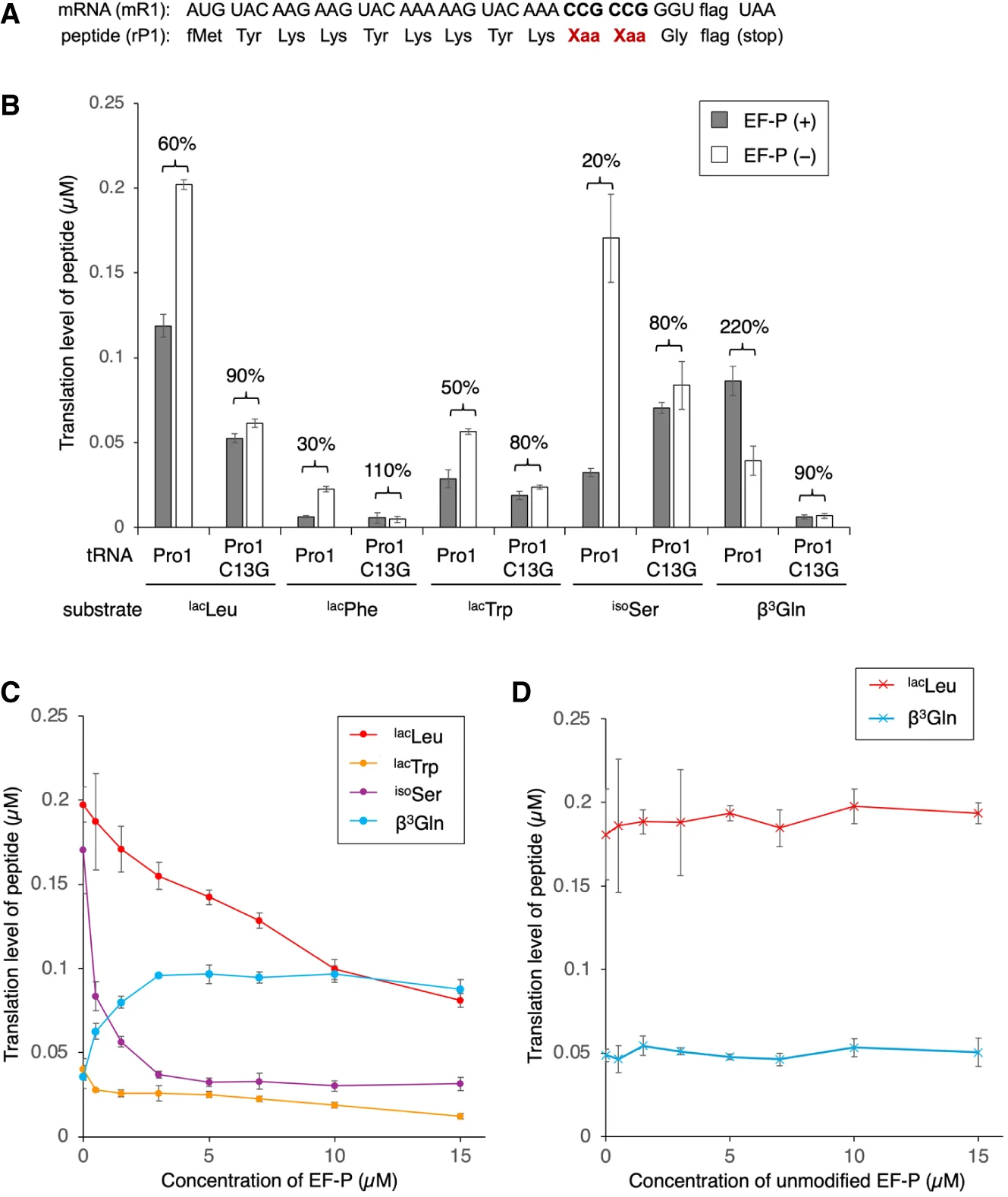
Ribosomal incorporation of α-hydroxy acids into model peptides and its inhibition by EF-P. (*A*) The mRNA sequence (mR1) and the corresponding peptide sequence (rP1) used in this experiment. The amino acid sequence of the FLAG tag is Asp-Tyr-Lys-Asp-Asp-Asp-Asp-Lys. (*B*) Translation levels of rP1 in which two consecutive α-hydroxy acids or β-amino acids were introduced at CCG codons using either tRNA^Pro1^_CGG_ or tRNA^Pro1C13G^_CGG_. Gray bars indicate EF-P (+) translation; white bars, EF-P (−) translation. Numbers above the bars represent the relative translation level, expressed as a percentage of the yield with EF-P relative to that without EF-P. EF-P concentration in EF-P (+) translation was 5 μM. Error bars, SD (*n* = 3). (*C*,*D*) EF-P titration experiments for ribosomal incorporation of two consecutive α-hydroxy acids or β-amino acids into rP1. EF-P bearing β-lysylation at Lys34 was used in *C*, whereas unmodified EF-P was used in *D*. Error bars, SD (*n* = 3).

In this study, the ribosomal incorporation efficiencies of four α-hydroxy acids—isopropyllactic acid (^lac^Leu), phenyllactic acid (^lac^Phe), indolelactic acid (^lac^Trp), and isoserine (^iso^Ser)—were assessed in the presence of EF-P (Fig. 1). In addition, (*S*)-β^3^-homoglutamine (β^3^Gln) was also evaluated as a positive control. EF-P has been previously shown to markedly enhance the ribosomal incorporation of β^3^Gln (Katoh and Suga 2018). Each substrate was precharged onto tRNA^Pro1^_CGG_, which bears a CGG anticodon (Fig. 2A), using flexizyme, an aminoacylation ribozyme (Goto et al. 2011; Katoh and Suga 2024). These were then introduced at two consecutive CCG codons in the mRNA template mR1 to direct the synthesis of a model peptide/ester hybrid, rP1 (Fig. 3A). Translation was performed at 37°C for 10 min using the flexible in vitro translation (FIT) system, either in the presence or absence of 5 µM EF-P [Fig. 3B, EF-P (+) and EF-P (−), respectively] (Yamagishi et al. 2011). The FIT system was supplied with [^14^C]-labeled Asp, unlabeled Gly, Lys, Met, and Tyr, and their corresponding ARSs, while all other amino acid/ARS pairs were omitted.

rP1 translation levels were assessed by tricine SDS–PAGE followed by autoradiography (Fig. 3B; Supplemental Fig. S2A). As expected, the yield of rP1 containing two β^3^Gln residues (rP1-β^3^Gln) increased 2.2-fold in the presence of EF-P. In contrast, the incorporation of α-hydroxy acids was markedly reduced, with yields reduced to 60%, 30%, 50%, and 20% for ^lac^Leu, ^lac^Phe, ^lac^Trp, and ^iso^Ser, respectively, relative to the yields obtained in the absence of EF-P. These results reveal an EF-P-mediated inhibition of α-hydroxy acid incorporation. Product identities were verified by MALDI-TOF MS analysis of translation reactions performed with unlabeled Asp (Supplemental Fig. S1), which confirmed the presence of only the expected products.

Because EF-P recognizes a specific D-arm motif in the P-site peptidyl-tRNA to facilitate peptidyl transfer, I investigated whether this motif is also required for the observed inhibition. A C13G mutation in the D-arm of tRNA^Pro1^ is known to disrupt EF-P-mediated promotion (Fig. 2A; Katoh et al. 2016). Therefore, the four α-hydroxy acids (^lac^Leu, ^lac^Phe, ^lac^Trp, and ^iso^Ser) and β^3^Gln were precharged onto the mutant tRNA, tRNA^Pro1C13G^_CGG_, and tested for incorporation into rP1, either in the presence or absence of 5 µM EF-P (Fig. 3B). Compared with the original tRNA^Pro1^_CGG_, both the EF-P-mediated inhibition of α-hydroxy acid incorporation and the promotion of β^3^Gln incorporation were attenuated (Fig. 3B; relative yields of 90%, 110%, 80%, 80%, and 90% for ^lac^Leu, ^lac^Phe, ^lac^Trp, ^iso^Ser, and β^3^Gln, respectively). These findings demonstrate that EF-P must recognize the specific D-arm motif to exert both its inhibitory and stimulatory effects.

### Dominant ester bond formation by ^iso^Ser

Among the four α-hydroxy acids examined, ^iso^Ser is unique as it is both a β^2^-amino acid and an α-hydroxy acid, possessing an amino group at the β-carbon and a hydroxy group at the α-carbon (Fig. 1A). Consequently, when ^iso^Ser is at the A-site, either its β-amino or α-hydroxyl group could, in principle, react with the carbonyl carbon of the P-site peptidyl-tRNA to form an amide or an ester bond, respectively. Given that EF-P typically promotes β-amino acid incorporation but inhibits α-hydroxy acid incorporation, our observation that ^iso^Ser incorporation is reduced by EF-P suggests that ester bond formation via the α-hydroxy group predominates over amide bond formation via the β-amino group. To confirm that β^2^-amino acid incorporation is indeed promoted by EF-P, β^2^Ala—a derivative of ^iso^Ser in which the α-hydroxy group is replaced by a methyl group—was tested in the presence and absence of 5 µM EF-P (Supplemental Fig. S3). Because the consecutive incorporation of two β^2^Ala residues into rP1 was too inefficient for quantitative analysis, its single incorporation into rP1′ was evaluated. The yield of rP1′-β^2^Ala increased 1.5-fold with EF-P (Supplemental Fig. S3B), consistent with EF-P-mediated promotion of peptide bond formation. In contrast, ^iso^Ser incorporation into rP1 was strongly inhibited by EF-P, resulting in a yield of only 20% relative to the reaction without EF-P (Fig. 3B), further supporting the conclusion that ester bond formation is the dominant pathway for this substrate.

### Titration of EF-P concentration in α-hydroxy acid incorporation

To more precisely assess the inhibitory and stimulatory effects of EF-P, EF-P concentration titrations were performed during rP1 synthesis incorporating either α-hydroxy acids or β^3^Gln using tRNA^Pro1^_CGG_ (Fig. 3C). Increasing the EF-P concentration from 0 to 15 µM led to progressive decreases in the yields of rP1 containing ^lac^Leu, ^lac^Trp, and ^iso^Ser (Fig. 3C; from 0.20 to 0.08 µM for ^lac^Leu, 0.04 to 0.01 µM for ^lac^Trp, and 0.17 to 0.03 µM for ^iso^Ser). In contrast, the yield of rP1-β^3^Gln increased with EF-P addition and plateaued at concentrations ≥3 µM (Fig. 3C; from 0.04 to 0.10 µM). At concentrations above 3 µM, EF-P likely remains bound to the ribosomal E site after peptidyl transfer, potentially impeding the translocation of deacylated tRNA from the P site to the E site. Under such conditions, EF-P may no longer promote—and could even inhibit—further peptidyl transfer. Similar titration patterns have been observed in our previous studies involving the consecutive β^3^-amino acids, d-amino acids, and l-Pro (Katoh et al. 2016, 2017; Katoh and Suga 2018).

Notably, while EF-P strongly inhibited ^iso^Ser incorporation—indicating predominant ester bond formation via the α-hydroxy group—the yield did not decrease further at EF-P concentrations above 5 µM, unlike other α-hydroxy acids. Instead, the incorporation level plateaued (Fig. 3C), suggesting that amide bond formation via the β-amino group also occurred to some extent. This minor pathway was likely promoted, rather than inhibited, by EF-P, thereby competing with ester bond formation. It has been reported that β-amino-α-hydroxy acids, such as ^iso^Ser, can be ribosomally incorporated through ester bond formation at the α-hydroxy group, followed by an *O*-to-*N* acyl shift to yield an amide bond (Kuroda et al. 2022). Thus, even if ^iso^Ser is initially incorporated via an ester linkage, the final product would be an amide.

It is well established that EF-P requires β-lysylation at Lys34 to facilitate peptidyl transfer (Doerfel et al. 2013; Ude et al. 2013). Accordingly, β-lysylated EF-P was used throughout this study unless otherwise specified. To examine whether β-lysylation is required for the inhibition of α-hydroxy acid incorporation, unmodified EF-P was tested. Specifically, unmodified EF-P was titrated during the incorporation of ^lac^Leu and β^3^Gln using tRNA^Pro1^_CGG_ (Fig. 3D). No significant changes in rP1 yields were observed for either ^lac^Leu or β^3^Gln across the 0–15 µM range, indicating that β-lysylation is essential for both the inhibition of α-hydroxy acid incorporation and the promotion of β^3^-amino acid incorporation.

### Ribosomal synthesis of model peptides containing β-amino and d-amino acids as well as α-hydroxy acids

**FIGURE 4.**
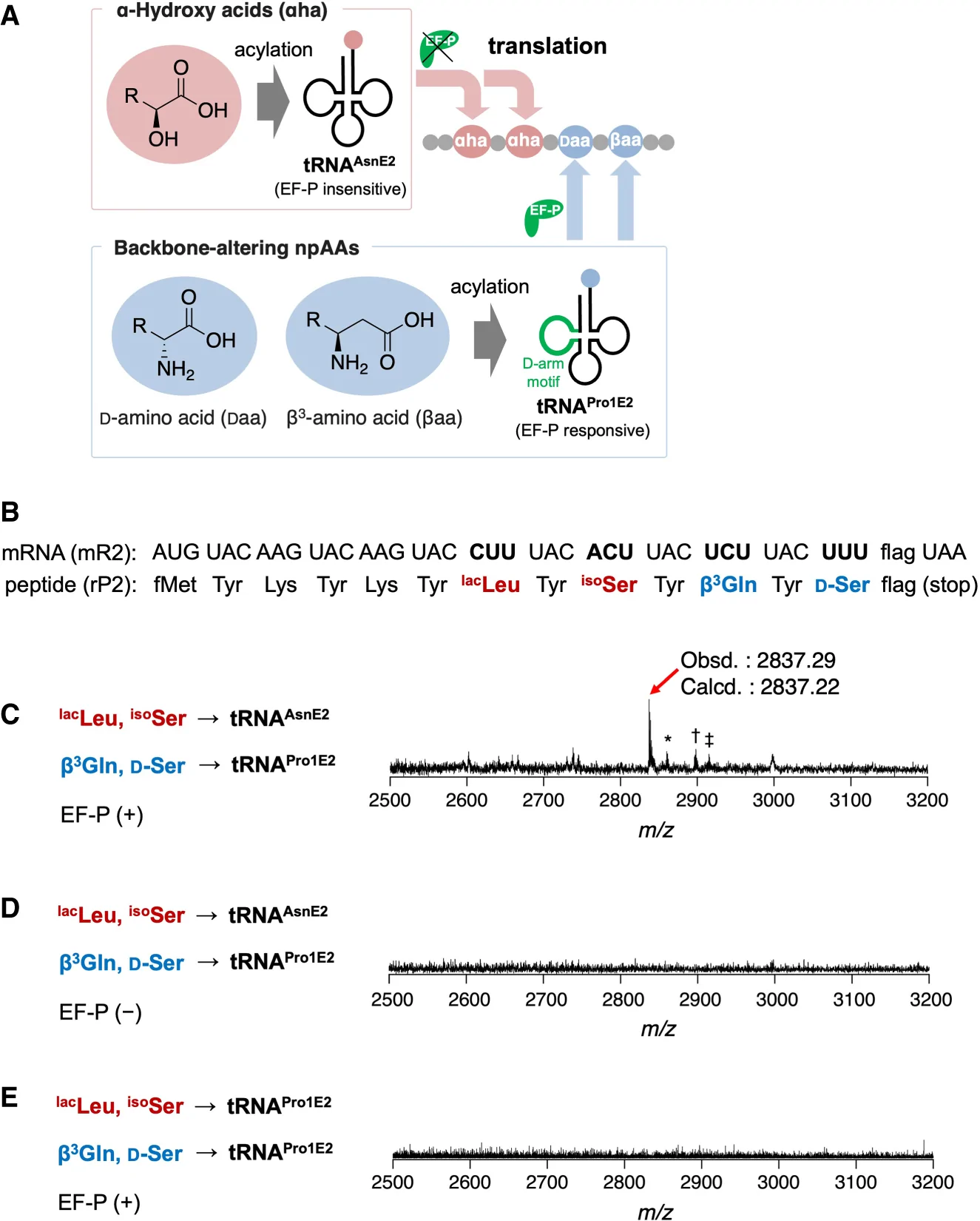
Simultaneous ribosomal incorporation of α-hydroxy acid, β-amino acid, and d-amino acid into a model peptide using engineered tRNAs in the presence of EF-P. (*A*) Overview of the dual tRNA strategy: charging α-hydroxy acids onto EF-P-insensitive tRNA^AsnE2^ while using tRNA^Pro1E2^ for npAAs. This enables the successful EF-P-dependent synthesis of complex depsipeptides. (*B*) Sequences of model mRNA (mR2) and the corresponding peptide (rP2). (*C*–*E*) MALDI-TOF MS analysis of translated rP2, in which ^lac^Leu, ^iso^Ser, β^3^Gln, and d-Ser were introduced using tRNA^AsnE2^ or tRNA^Pro1E2^ in the presence or absence of EF-P. The red arrow in *C* indicates the peak corresponding to the desired product. “Obsd.” and “Calcd.” denote observed and calculated *m/z* values of the desired product, respectively. (*) Na^+^ adduct of the desired product. (^†^) Phe misincorporation in place of d-Ser. (^‡^) Na^+^ adduct of the Phe misincorporation byproduct.

Engineered tRNAs, such as tRNA^Pro1E2^, enable the ribosomal incorporation of otherwise inefficient backbone-altering npAAs—including β-amino and d-amino acids—when used in combination with EF-P. However, the simultaneous introduction of α-hydroxy acids alongside these npAAs is challenging. Given that EF-P inhibits their incorporation when charged onto tRNAs bearing the EF-P-responsive D-arm motif, tRNA^Pro1E2^ is unsuitable for α-hydroxy acids. To facilitate β-/d-amino acid incorporation via EF-P while preserving the incorporation efficiency of α-hydroxy acids, a dual-tRNA strategy was employed: tRNA^AsnE2^—an orthogonal tRNA lacking the EF-P-responsive D-arm motif—was used for α-hydroxy acids, while tRNA^Pro1E2^ was used for β-/d-amino acids (Fig. 4A). tRNA^AsnE2^ is an engineered derivative of *E. coli* tRNA^Asn^ that has been previously utilized for the incorporation of various npAAs, including α-hydroxy acids (Ohta et al. 2007).

Using this approach, ^lac^Leu, ^iso^Ser, β^3^Gln, and d-Ser were introduced into the model peptide rP2 at the CUU, ACU, UCU, and UUU codons of mRNA mR2, respectively (Fig. 4B). Two tRNA body combinations were compared: (1) tRNA^AsnE2^ for ^lac^Leu and ^iso^Ser, and tRNA^Pro1E2^ for β^3^Gln and d-Ser; and (2) tRNA^Pro1E2^ for all four substrates (Fig. 4C–E). In the presence of EF-P, combination (1) successfully yielded rP2 as the primary peak in MALDI-TOF/MS analysis, although a minor byproduct peak from Phe misincorporation at the UUU codon (instead of ^iso^Ser) was detected (Fig. 4C). This misincorporation is likely due to trace Phe-tRNA^Phe^ contamination. In the absence of EF-P, no rP2 product was observed for combination (1) (Fig. 4D), consistent with the low incorporation efficiency of β^3^Gln and d-Ser without EF-P. This confirms that EF-P-assisted incorporation of these two npAAs is essential for rP2 synthesis. In contrast, combination (2) produced no detectable rP2 even in the presence of EF-P (Fig. 4E). This failure is likely because EF-P inhibited the incorporation of ^lac^Leu and ^iso^Ser when they are charged on tRNA^Pro1E2^, reducing the overall translation efficiency to undetectable levels. These results demonstrate that the strategic selection of tRNA bodies is critical for the co-incorporation of α-hydroxy acids and other backbone-altering substrates in EF-P-assisted translation.

## DISCUSSION

This study revealed that EF-P inhibits the ribosomal incorporation of four α-hydroxy acids when they are charged onto EF-P-responsive tRNAs bearing the specific D-arm motif, such as tRNA^Pro1^ and tRNA^Pro1E2^. Previously, I reported that while the incorporation of β-amino acids is generally promoted by EF-P (Katoh and Suga 2018), certain variants with constrained cyclic backbone structures are exceptionally inhibited (Katoh et al. 2020). For example, the incorporation of (1*R*,2*S*)-2-aminocyclopentanecarboxylic acid (Acpc) is enhanced, whereas that of (1*S*,2*S*)-2-Acpc is suppressed, indicating that the effect of EF-P on β-amino acid incorporation is sensitive to both the stereochemistry and backbone conformation of the substrate. Notably, although the α-hydroxy acids tested in this study share the same stereochemistry and backbone scaffold as canonical l-α-amino acids—differing only in the nucleophilic group—their incorporation is significantly inhibited by EF-P. It is plausible that while most β-amino acids and (1*R*,2*S*)-2-Acpc are positioned favorably for peptidyl transfer in the presence of EF-P, α-hydroxy acids and (1*S*,2*S*)-2-Acpc adopt less favorable orientations that hinder the reaction.

Mechanistically, EF-P is known to facilitate the peptidyl transfer of Pro-Pro sequences through entropic steering, orienting the P-site peptidyl-prolyl-tRNA into a catalytically productive conformation (Doerfel et al. 2015; Huter et al. 2017). Recent structural and computational studies on diverse noncanonical monomers suggest that efficient bond formation requires the P-site electrophilic carbonyl to be positioned within a specific reactive proximity (<4 Å) and Bürgi–Dunitz angle (76°–115°) relative to the A-site nucleophile (Watson et al. 2023; Hamlish et al. 2024). I hypothesize that while EF-P effectively steers the P-site carbonyls of canonical (Pro) or certain npAA substrates (e.g., β-amino and d-amino acids) into such reactive orientations, it may conversely reposition α-hydroxy acids—which might otherwise occupy a relatively favorable orientation—into suboptimal conformations, thereby hindering efficient bond formation. High-resolution structural analysis of the ribosome-EF-P-hydroxyacyl-tRNA complex will be essential to confirm this model.

Ribosomally synthesized bioactive peptides containing npAAs provide a powerful platform for developing novel peptide therapeutics, as random peptide libraries generated by ribosomal synthesis can be directly integrated into mRNA display-based screening systems, such as the Random nonstandard Peptide Integrated Discovery (RaPID) method (Nemoto et al. 1997; Roberts and Szostak 1997; Katoh et al. 2022). By utilizing tRNA^Pro1E2^, we have constructed peptide libraries incorporating diverse npAAs—including β-amino, γ-amino, and d-amino acids—and successfully identified bioactive peptides via RaPID selection (Katoh et al. 2020; Imanishi et al. 2021; Katoh and Suga 2022; Wakabayashi et al. 2022; Miura et al. 2023, 2024a,b). However, as demonstrated here, EF-P inhibits α-hydroxy acid incorporation when using tRNA^Pro1E2^, limiting their inclusion in EF-P-assisted translation. To overcome this limitation, a combination of EF-P-insensitive and EF-P-responsive tRNAs can be employed to co-introduce α-hydroxy acids alongside other npAAs. This strategy enables the simultaneous incorporation of multiple classes of npAAs into a single peptide library, facilitating the discovery of novel polypeptide/polyester hybrids—depsipeptides—containing a diverse array of noncanonical building blocks.

## MATERIALS AND METHODS

### Preparation of tRNAs and flexizymes

tRNAs and flexizymes (dFx and eFx) used for acyl-tRNA preparation were synthesized in vitro by T7 RNA polymerase transcription. Template DNAs were generated by primer extension using forward and reverse extension primers, followed by PCR amplification with forward and reverse PCR primers (Supplemental Table S1 for sequences). For tRNA templates, the second nucleotide from the 5′-end in the reverse PCR primer was modified with 2′-*O*-methylation. Extension reactions were carried out at 95°C for 1 min, followed by five cycles of 50°C for 1 min and 72°C for 1 min, in a reaction mixture containing 10 mM Tris-HCl (pH 9.0), 50 mM KCl, 0.1% (v/v) Triton X-100, 2.5 mM MgCl_2_, 250 μM dNTP mix, 60 nM *Taq* DNA polymerase, and 2 μM each of forward and reverse extension primers. PCR amplification was then performed for 15 cycles of 95°C for 40 sec, 50°C for 40 sec, and 72°C for 40 sec in the same buffer supplemented with 0.5 μM forward and reverse PCR primers and 1/100 vol. of the extension reaction as a template. The resulting PCR products were purified by phenol–chloroform extraction followed by ethanol precipitation. Transcription of tRNAs and flexizymes was performed at 37°C for 16 h in 0.5–4 mL reaction mixtures containing 40 mM Tris-HCl (pH 8.0), 22.5 mM MgCl_2_, 1 mM dithiothreitol (DTT), 1 mM spermidine, 0.01% Triton X-100, 3.75 or 5 mM nucleoside triphosphate (NTP) mix, 0.04 U/µL RNasin RNase inhibitor (Promega M6101), 0.12 µM T7 RNA polymerase, and the corresponding PCR product. The NTP concentration was 3.75 mM for tRNA synthesis and 5 mM for flexizyme synthesis. For tRNA preparation, 5 mM GMP or CMP was added depending on the 5′-terminal nucleotide (GMP for tRNA^Pro1E2^ and tRNA^AsnE2^, CMP for tRNA^Pro1^). The resulting RNAs were treated with RQ1 DNase (Promega) at 37°C for 30 min and purified by denaturing polyacrylamide gel electrophoresis containing 6 M urea (8% gel for tRNA, 12% gel for flexizyme).

### Preparation of acyl-tRNAs

Preactivated hydroxy/amino acids—^lac^Leu-3,5-dinitrobenzyl ester (DBE), ^lac^Phe-cyanomethyl ester (CME), ^lac^Trp-CME, ^iso^Ser-DBE, β^2^Ala-DBE, β^3^Gln-DBE, and d-Ser-DBE—were synthesized as previously described (Saito et al. 2001; Murakami et al. 2006; Ohta et al. 2007). Acylation reactions were performed at 0°C in mixtures containing 50 mM HEPES-KOH (pH 7.5 or 8.0) or 50 mM bicine-KOH (pH 8.7), 600 mM MgCl_2_, 20% DMSO, 25 µM dFx or eFx, 25 µM tRNA, and 5 mM preactivated hydroxy/amino acid. Reaction pH was adjusted to 7.5 for d-Ser, 8.0 for ^lac^Leu, ^lac^Phe, ^lac^Trp, ^iso^Ser, and β^2^Ala, and 8.7 for β^3^Gln. dFx was used for DBE-activated substrates and eFx for CME-activated ones. Reaction times were 3 h for ^lac^Leu, ^lac^Phe, and ^lac^Trp; 6 h for d-Ser; 16 h for ^iso^Ser; and 22 h for β^2^Ala and β^3^Gln. The resulting acyl-tRNAs were recovered by ethanol precipitation, washed twice with 70% ethanol containing 0.1 M sodium acetate (pH 5.2), followed by one wash with 70% ethanol, and dissolved in 1 mM sodium acetate (pH 5.2).

### Expression and purification of *E. coli* EF-P

*E. coli* EF-P was expressed using a pET28a vector carrying the EF-P gene (see our previous report for vector construction) (Katoh et al. 2016). For β-lysylation of EF-P, the modification enzymes EpmA and EpmB were cloned into a pETDuet-1 vector. EF-P/pET28a and EpmA/EpmB/pETDuet-1 were co-transformed into *E. coli* Rosetta2 (DE3). To produce EF-P lacking β-lysylation, the EpmA/EpmB/pETDuet-1 vector was omitted. Cells were cultured at 37°C in LB medium for 2 h without IPTG, followed by induction with 0.5 mM IPTG for 2 h. The harvested cells were lysed by sonication, and lysates were centrifuged at 7000*g* for 10 min. The supernatant was applied to a HiTrap TALON crude 5 mL column (Cytiva), washed with buffer A (20 mM Tris-HCl [pH 8.0], 200 mM NaCl, 20 mM imidazole, and 1 mM DTT), and eluted by increasing the imidazole concentration in buffer A to 300 mM. Turbo3C protease was added to the eluate to cleave the His-tag, and the sample was dialyzed against buffer A at 4°C overnight. The dialyzed sample was reapplied to the HiTrap TALON column, and the flowthrough and wash fractions were collected to obtain EF-P lacking the His-tag.

### In vitro translation of model peptides

Translations were carried out at 37°C for 10 min in 2.5 µL FIT system reactions containing 50 mM HEPES-KOH (pH 7.6), 100 mM potassium acetate, 12.3 mM magnesium acetate, 2 mM ATP, 2 mM GTP, 1 mM CTP, 1 mM UTP, 20 mM creatine phosphate, 0.1 mM 10-formyl-5,6,7,8-tetrahydrofolic acid, 2 mM spermidine, 1 mM DTT, 1.5 mg/mL *E. coli* total tRNA, 1.2 µM *E. coli* ribosome, 0.6 µM methionyl-tRNA formyltransferase, 2.7 µM IF1, 3 µM IF2, 1.5 µM IF3, 0.1 µM EF-G, 20 µM EF-Tu/Ts, 0.25 µM RF2, 0.17 µM RF3, 0.5 µM RRF, 4 µg/mL creatine kinase, 3 µg/mL myokinase, 0.1 µM inorganic pyrophosphatase, 0.1 µM nucleotide diphosphate kinase, 0.1 µM T7 RNA polymerase, 0.1 µM AspRS, 0.1 µM GlyRS, 0.2 µM LysRS, 0.1 µM MetRS, 0.1 µM TyrRS, 0.05 mM [^14^C]-aspartic acid, 0.5 mM glycine, 0.5 mM lysine, 0.5 mM methionine, 0.5 mM tyrosine, 20 µM of each precharged acyl-tRNA, and 2 µM DNA template. Reactions were quenched by adding an equal volume of 2× stop solution [0.9 M Tris-HCl (pH 8.45), 8% SDS, 30% glycerol, and 0.001% xylene cyanol] and incubating at 95°C for 2 min. Samples were analyzed by 15% tricine SDS–PAGE followed by autoradiography using a Typhoon FLA 7000 (Cytiva). Translation yields were quantified based on the autoradiographic intensity of the [^14^C]-labeled peptide bands. For MALDI-TOF mass spectrometry, [^14^C]-aspartic acid was replaced with unlabeled aspartic acid. Translation products were desalted using SPE C-tip (Nikkyo Technos) and eluted with 0.8 μL of 80% acetonitrile, 0.5% acetic acid containing 50%-saturated (*R*)-cyano-4-hydroxycinnamic acid. Samples were analyzed on an UltrafleXtreme (Bruker Daltonics) in reflector/positive mode, with peptide calibration standard II (Bruker Daltonics) used for external mass calibration.

## SUPPLEMENTAL MATERIAL

Supplemental material is available for this article.
